# Nanoparticles as a Solution for Eliminating the Risk of Mycotoxins

**DOI:** 10.3390/nano8090727

**Published:** 2018-09-14

**Authors:** Pavel Horky, Sylvie Skalickova, Daria Baholet, Jiri Skladanka

**Affiliations:** Department of Animal Nutrition and Forage Production, Faculty of AgriSciences, Mendel University, 61300 Brno, Czech Republic; pavel.horky@mendelu.cz (P.H.); xbaholet@mendelu.cz (D.B.); jiri.skladanka@mendelu.cz (J.S.)

**Keywords:** mycotoxin, nanotechnology, agriculture, toxicity, nanoparticles

## Abstract

Mycotoxins are toxic secondary metabolites produced by certain filamentous fungi. The occurrence of mycotoxins in food and feed causes negative health impacts on both humans and animals. Clay binders, yeast cell walls, or antioxidant additives are the most widely used products for mycotoxin elimination to reduce their impact. Although conventional methods are constantly improving, current research trends are looking for innovative solutions. Nanotechnology approaches seem to be a promising, effective, and low-cost way to minimize the health effects of mycotoxins. This review aims to shed light on the critical knowledge gap in mycotoxin elimination by nanotechnology. There are three main strategies: mold inhibition, mycotoxin adsorption, and reducing the toxic effect via nanoparticles. One of the most promising methods is the use of carbon-based nanomaterials. Graphene has been shown to have a huge surface and high binding capacity for mycotoxins. Attention has also been drawn to polymeric nanoparticles; they could substitute adsorbents or enclose any substance, which would improve the health status of the organism. In light of these findings, this review gives new insights into possible future research that might overcome challenges associated with nanotechnology utilization for mycotoxin elimination from agricultural products.

## 1. Introduction

Mycotoxins are produced by molds under specific conditions, such as high humidity, poor agricultural practices, or damaged and contaminated crops. Although the presence of molds on grains does not necessarily mean there are mycotoxins present, the potential for mycotoxin production does exists. Further, the long-term absence of molds on stored food and feed does not guarantee that the grain is free of mycotoxins [1,2]. The issue of mycotoxin risk is therefore tricky and requires the attention of both agrotechnology specifically and the scientific community generally.

The main producers of mycotoxins are species of *Aspergillus* (aflatoxins (AFL), ochratoxin A (OTA), trichothecenes, and deoxynivalenol (DON)), *Fusarium* (zearalenone (ZEA), fumonisins (FUM) B1 and B2, and the emerging mycotoxins fusaproliferin, moniliformin, beauvericin, and enniatins), *Claviceps* (ergot alkaloids), and *Alternaria* (altenuene, alternariol, alternariol methyl ether, altertoxin, and tenuazonic acid) [3]. These mycotoxins are among the most dangerous. They may cause cardiotoxicity, central nervous system disorders, gastrointestinal tract damage, nephrotoxicity, and hepatotoxicity. The mechanisms of mycotoxin toxicity have been studied for many years [4,5,6,7,8,9]. Currently, there are about 500 species of mycotoxins and it is estimated that another 1000 have yet to be discovered. Especially, masked mycotoxins pose a great risk because there is no established routine method for determining them [10]. 

Correct crop management practices play an important role in mycotoxin occurrence prevention. Furthermore, during the growing period, negative impacts on mycotoxin propagation could include drought, insect attack, temperature fluctuations, or crop rotation [11]. Humidity, grain fragments, or weeds are the major factors for mold propagation in stored materials [12]. 

Mycotoxins are known to cause a number of toxic effects in animal species. The most sensitive is poultry, followed by pigs and ruminants [13]. Generally, it is well known that ruminants metabolize some kinds of mycotoxins with almost 100% efficacy [14]. Mycotoxins have the ability to become part of animal products because they are largely lipophilic [15]. For example, AFL M1 easily gets into milk, which can cause serious health problems [16]. 

Recent trends in mycotoxin elimination from food and feed have led to the application of various adsorbents as nutritional additives. The most used are clay particles such as bentonites and zeolites due to their opposite polarity [17]. The disadvantage of clay absorbers is their ability to bind to minerals and vitamins from feed. For this reason, the dietary content of micronutrients have to be increased by an average of 20% [18,19]. Besides, clay adsorbents could be used in yeast cell walls (*Saccharomyces cerevisiae*) [20,21,22]. However, the effectiveness of mycotoxin absorbents themselves ranges from 20% to 80%. A single assessment of the effectiveness of adsorbents has not yet been established. 

Nevertheless, mycotoxins are an important issue in a number of disciplines such as food science, toxicology, applied and analytical chemistry, veterinary sciences, mycology, plant science, and agriculture. This topic has been studied far less from a nanotechnological point of view, although nanotechnology is a dynamically evolving discipline [23,24]. The key properties in this field of research are based on the nanoscale. For example, gravity is no longer relevant here as well as the shape and net charge, which greatly changes the behavior of the material. Nanotechnologies allow for the alteration of already discovered properties and the creation of endlessly new materials with exciting possibilities [25]. Nowadays, some of the latest developments in nanotechnology are commercially used in medicine, the household, textiles, and electrotechnics. 

In this review, we focus on the key words mycotoxins, nanotechnology, nanoparticles (NPs), and agriculture. Recent research suggests that nanotechnology is increasingly penetrating the field of agriculture and mycotoxins. The first development of nanotechnological applications for mycotoxin elimination or detection have been implemented since 2009 [26]. Although several studies have already focused on the use of nanotechnology for mycotoxin elimination, these experimental results have not yet been reviewed.

## 2. Mycotoxin Detection Using Nanoparticles

Regarding mycotoxins, many analytical methods have been developed for their reliable determination. Currently, the most commonly used approaches in practice are high-pressure liquid chromatography (HPLC) and mass detection and enzyme-linked immunoassay (ELISA) [27,28]. Since early detection is needed to protect health, current research has focused on improving the detection limit, time consumption, sample consumption, and ease of use [29,30,31,32]. Practice distinguishes two different types of NP utilization in detection systems [33,34]. On the receptor level, NPs directly react with the detected molecule. This system requires adequate specificity and reproducibility. NPs could be evolved as a transducer-enhancing signal to the detector. An overview of these technologies is given by Rai et al. (2015). This work summarizes the possibilities of the immobilization of biomolecules and states that mycotoxins warrant further research regarding the construction of nanobiosensors with more stability and durability [33]. In this case, the advantage of NPs is their high surface-area-to-volume ratio, which enables the binding of higher concentrations of mycotoxins [35]. Over the past decade, research in nanomaterials has focused on carbon nanotubes, polymers, superparamagnetic NPs, quantum dots, and metal NPs. In addition, the use of nanoparticles allows various modifications with the specific ligands or surface decoration by functional groups such as CH_3_, −OH, −COOH, −NH_2_, or −CONH_2_. 

**Figure 1:**
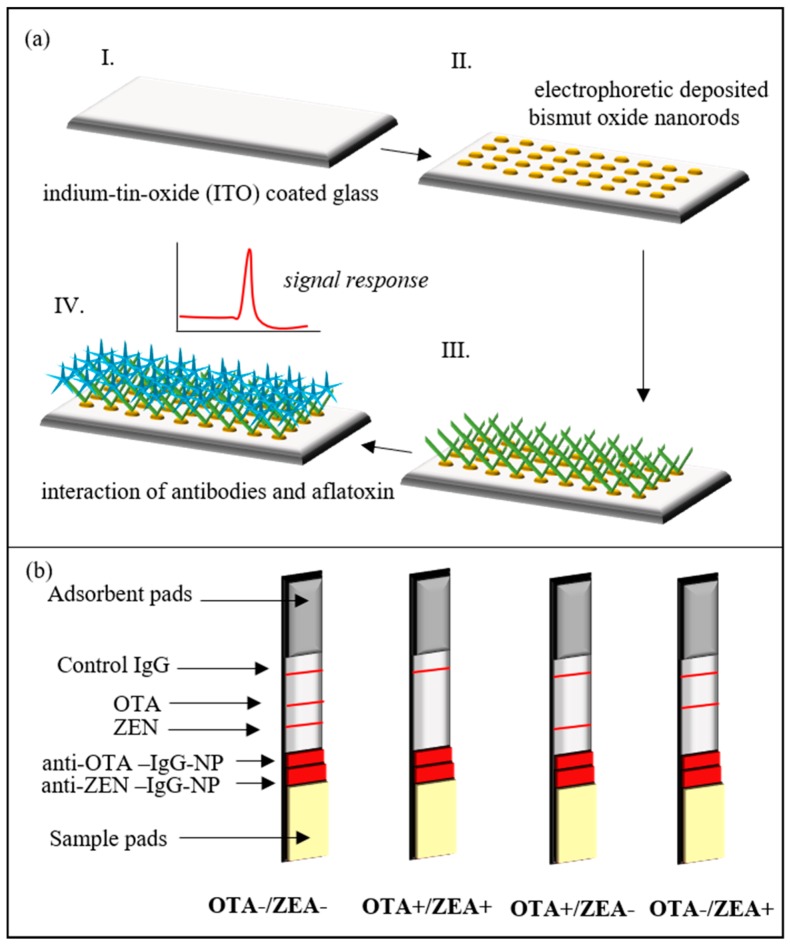
Different immunodetection arrangements. (**a**) An electrosensor based on the interaction of the antibody with mycotoxin, which amplifies the signal; (**b**) A lateral easy-to-use immunoassay for the detection of mycotoxins by the naked eye. Figures adapted from [37,42], with permission from Elsevier (2017) and John Wiley and Sons (2015), respectively.

Much of the current literature on mycotoxin detection pays particular attention to immunodetection of mycotoxins (Figure 1). Lateral flow immunochromatographic assay is a rapidly developing technique which combines antibodies (for specificity) and NPs (for sensitivity). Taking advantage of gold NPs of quantum dots, the limit of detection for various mycotoxins is in the range of 0.1–10,000 ng/mL [36]. Immunoelectrodes have been designed based on bismuth oxide nanorods for AFL B1 detection [37] (Figure 1a). The third generation of immunosensors is characterized by rapid response (15 s), high sensitivity (1.132 μA/(ng/dL)), broad linear range (1–70 ng/dL), and low detection limit (8.715 ng/dL) and operates by direct electron transfer of analytes to electrodes [38]. Immunochromatographic ready-to-use test strips have recently been proposed for the simultaneous detection of ZEA and T2 toxin with detection limits of 0.1 and 0.05 ng/mL. Perfect sensitivity and rapid testing have been achieved using antibody-labeled magnetic NPs for sample pretreatment [39]. Multilayer NPs can perform several functions simultaneously. (Lv et al.) (2017) designed RuSi@Ru(bpy)3(2+) loaded with gold-functioned nanoporous CO/Co_3_O_4_ electrochemiluminescence biosensors for sensitivity (5 pg/mL) detection of DON [40]. Ag@Au Core-Shell NPs have been used for surface-enhanced Raman scattering aptasensors for the double detection of OTA A and AFL B1. Principally, Raman scattering aptasensors produce stable and quantitative signals that emerge from the plasmonic coupling at the junction of a silver core and a gold shell [41]. The latest development has focused on one-step multiplex detection of various mycotoxins. Whether in the form of a strip or whatever form is needed for a device, this simplifies the whole analysis [42] (Figure 1b). Kong et al. made semiquantitative and quantitative multi-immunochromatographic strips based on gold NPs as a label for the detection of 20 mycotoxin. An advantage of this is the ability to read results by the naked eye. The visual limits of detection for ZEAs, DONs, T2s, AFs, and FUMs were estimated to be 0.1–0.5, 2.5–250, 0.5–1, 0.25–1, and 2.5–10 μg/kg, respectively [43]. 

The abovementioned findings demonstrate the great potential of NPs for mycotoxin detection. Therefore, detection requirements are greater than sensitivity and reliability. Mycotoxin derivates are commonly undetectable by conventional analytical techniques due to their changed structure by plant enzymes [44]. Enzymatic or acid hydrolysis is often employed prior to mycotoxin determination as an effective pretreatment step of masked derivates [45]. Also, mass spectrometry could expose masked mycotoxins [46,47,48]. The design of a proof of concept for the identification of unknown modified masked mycotoxins is still a big challenge, which could be facilitated with NPs for both mycotoxin isolation or detection.

## 3. Nano Approaches for Mycotoxin Risk Elimination 

Recently, questions have been raised about the nanotechnology solution of mycotoxin risk. There are three general strategies: mold inhibition, mycotoxin adsorption, and impact elimination. 

### 3.1. Antifungal Nanomaterials Cause Mold Inhibition and Mycotoxin Production

The past decade has seen the rapid development of antibacterial nanoparticles as a solution for antibiotic resistance of pathogenic bacteria. Their applicability against mycotoxin occurrence has been limited by differences between bacteria and fungi. Bacteria are single celled, whereas most fungi are multicellular; bacteria have three distinct shapes, while fungi have various shapes which lead to mycelium formation; bacteria reproduce sexually, whereas fungi are capable of reproducing both sexually or asexually. All these differences make fungi more durable and resistant against some antibiotics [49]. Until now, research has tended to focus on antibacterial nanoparticles rather than on nanoparticles against fungi. The latest findings in the field of antifungal nanoparticles have been summarized between 2016 and 2017 [50,51,52,53,54]. In practice, the prevention of mycotoxin occurrence could be mediated via antifungal nanoparticles, which are easy to produce in large scale. According to recent scientific articles (summarized in Table 1 and Table 2), the antifungal strategy is oriented in two directions. Firstly, an antifungal compound is encapsulated into a polymeric nanocage. Perhaps the most serious disadvantage of this method is the instability in air, although nanopolymers allow cargo release under the appropriate conditions (e.g., presence of enzymes, higher temperature, pH change). Secondly, inhibition effect is reached by nanoparticles alone. This method mainly relies on metal nanoparticles which are stable, act immediately, and offer the possibility of green synthesis. Moreover, the advantage of green synthesis is the formation nanobiocomposites using plant, micro-organism, and animal sources which show less toxicity and improved their main features (Table 2) [55]. 

The relationship between an NP’s antifungal activity and mechanism of action has been investigated. He et al. studied the influence of ZnO nanoparticles on *Botrytis cinerea* and *Penicillium expansum*. ZnO NPs producing reactive oxide species (ROS) leads to the damage of the lipid bilayer cell membrane and the breakdown of the affected cell [74]. Scanning electron microscopy (SEM) showed the formation of unusual bulges on the surface of fungal hyphae and deformation of fungal hyphae after treatment with 12 mmol/L ZnO NPs. More recent attention has been focused on silver nanoparticles. The findings indicate that Ag NPs inhibit fungal growth as well as morphological and metabolic changes [85,86]. For instance, the application of 45 ppm Ag NPs caused a decrease of organic acid (oxalic, maleic, and citric acid) production, mycotoxin production (up to 80%), and changes in the enzymatic profile in *Aspergillus niger* and *Penicillium chrysogenum* [85]. All the studies reviewed so far, however, suffer from the fact that the interaction of NPs with the individual components of the fungi cells has not been investigated yet.

### 3.2. Nanoparticles Suitable for Mycotoxin Adsorption 

The high expectations of using nanomaterials as special adsorbents to remove pollutants relies not only on the high surface area and the high affinity to organic compounds (properties of conventional adsorbents such as those possessed by activated carbon) but also greatly on the fact that nanomaterials can be engineered or modified specifically to enhance selectivity to specific target pollutants [87]. Mycotoxins show a structural diversity resulting in different chemical and physical properties. Mycotoxins can be classified as polar or nonpolar molecules; however, there are several that fall in between. AFLs and FUMs are highly polar, while trichothecenes are polar and ZEAs are nonpolar [88,89]. This diversity could be resolved by such a material that changes its properties under various physicochemical conditions and can denote both polar and nonpolar substances [87]. The following subsections describe the most promising nanomaterials for the elimination of mycotoxins.

#### Carbon Nanostructures

**Figure 2:**
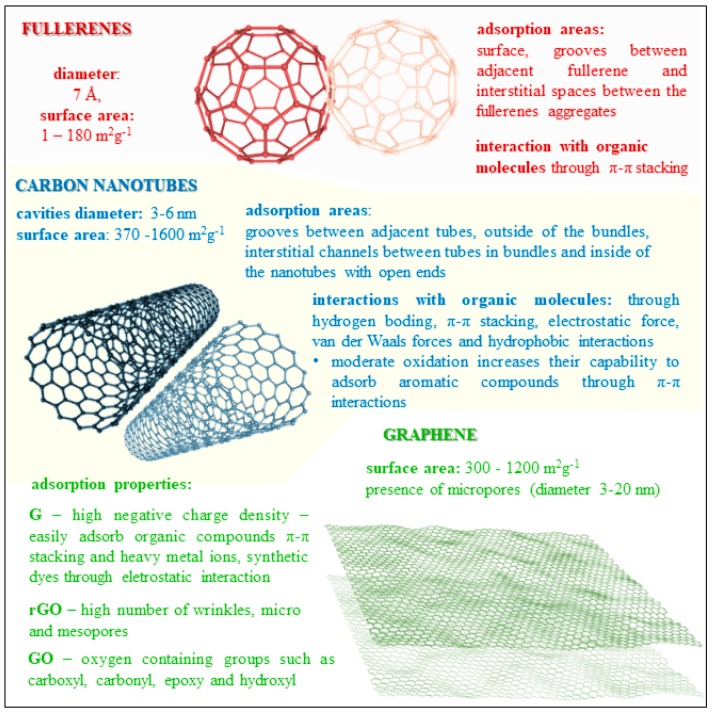
Key properties of carbon nanoparticles such as fullerenes, carbon nanotubes, and graphene (native graphene (G), reduced graphene (rGO), and graphene oxide (GO)). Mycotoxins can be bound to the surface, bundles, grooves, or channels between nanoparticles via different binding interactions.

Activated charcoal has been used for mycotoxin elimination for a long time. From this established practice proceeds the use of carbon nanoforms as a promising successor to activated carbon. The advantages of carbon nanomaterials are excellent stability, inertness, high adsorptive properties, large surface area per weight, and colloidal stability upon various pHs, which is important to preserve in the gastrointestinal tract [90]. Chemically, the carbon–carbon covalent bonds and crystalline structure provide specific properties such as strength, elasticity, and great conductivity. Graphene, graphene oxide, nanodiamonds, fullerenes, fiber, and nanotubes have a great potential to become novel adsorbents of mycotoxins. Nanocarbon structures are amphoteric and their surface could be protonated or deprotonated, which results in the binding capacity of polar or nonpolar compounds. Binding activity properties of carbon nanomaterials are summarized in Figure 2. Generally, carbon nanotube adsorption affinity poorly correlates with hydrophobicity but increases in the order of nonpolar aliphatic < nonpolar aromatics < nitroaromatics functional groups [91]. 

Fullerene adsorption behavior was found to be higher than that of activated carbon. Preliminary investigations of fullerenol C_60_(OH)_24_ nanoparticles on mycelial growth, aflatoxin production, and oxidative stress modulation in an aflatoxigenic strain of *Aspergillus flavus* showed slightly reduced mycelial biomass weight but significantly decreased aflatoxin concentration in media. A concentration 10 ng/mL of fullerenol reduced aflatoxin production compared to a control sample [92]. The possible mechanism could be explained by oxidative stress suppression of fungal culture [93]. 

Nanodiamonds show all the benefits of carbon nanomaterials and their large-scale production is considered to be inexpensive. Their chemical structure allows surface functionalization, including carboxylation, hydrogenation, and hydroxylation, which could provide binding affinity to various types of the mycotoxins. Nanodiamond aggregates (~40 nm) tend to adsorb AFL B1 and OTA via electrostatic interactions that depend on the types functional groups on the surface of nanodiamonds [94]. The adsorption capacity was estimated for AFL B1 as approximately 10 μg per mg of nanodiamonds and for OTA around 15 μg per mg of nanodiamonds. These results indicate greater adsorption capacities than commercially used yeast cell walls and clay minerals [95]. 

The perspective of single/multiwalled carbon nanotubes (CNT) should also be mentioned at this point. To date, CNTs are experimentally used for various medicinal applications such as drug administration, target therapy, or diagnostics in humans. In vivo experiments on mouse models indicated the absence of a pronounced toxic effect under conditions of a short-term experiment. On contrary, nanotubes affected mouse gastrointestinal mucosa and induced a weak immune response based on CNT modification and functionalization [96,97]. Nevertheless, their good adsorption capacity is well utilized in solid phase extraction protocols mainly for zearalenone [98,99,100], trichothecenes [101,102,103], and aflatoxins [104].

Magnetic graphene (MGO) synthesized from iron oxide nanostructures and graphene oxide is inexpensive and easily accessible. Numerous oxygen functional group on the MGO surface enable interaction with fusarium mycotoxins (DON, T1, and T2). An adsorption procedure was performed by dissolving palm kernel cake in water (6.2 pH for 5.2 h at 40.6 °C) and subsequently MGO with bounded mycotoxins was removed via magnet. Results showed that the reduction of mycotoxins varied from 40% to 70% for T2 and DON, respectively [105]. Surface active maghemite nanoparticles (SAMN) constituted of stoichiometric maghemite (γ-Fe_2_O_3_) showed chelating properties for citrinin and OTA toward iron(III) presence [106]. Monascus suspensions were treated with 1 gL^−1^ SAMNs, leading to 70% citrinin removal for the first time. A second treatment removed citrinin below the analytical detection limits (0.25 mg L^−1^). SAMNs represent an ideal material, as their synthetic protocol is suitable for being scaled up to an industrial level and is carried out in water without using of any organic solvent [106].

#### Chitosan Polymeric Nanoparticles

Chitosan (CS) is a natural cationic polysaccharide produced from chitin, which is the structural element found in the exoskeleton of crustaceans. In contrast to similar polysaccharide celluloses, CS contains hydroxyl groups, acetylamine, or free amino groups which has attracted attention in many fields of applications. CS is nontoxic, biodegradable, and possesses low immunogenicity. Therefore, CS has shown promising results for mycotoxin elimination from different raw materials. In 1990s, CS began to be considered as a suitable mycotoxin adsorbent with approximately 70% efficacy [107]. In addition, a CS solution in a mixture with the minerals rektorit and attapulgit has been patented for removing feed zearalenone and reducing diarrhea due to its antimicrobial properties [108].

Although carbon nanostructures are the focus of much research nowadays, chitosan polymer and nanoparticles have been highlighted in the years 2010–2015. CS is easily subjected to nanoparticles via the gelatation process using aldehydes (e.g., glutaraldehyde) and acids (e.g., thioglycolic, acrylic, and oxalic acids) [109]. Another way for nanoparticle formation is ionic cross links based on electrostatic interaction with phosphoric acid derivatives such as sodium tripolyphosphate (TPP) [110]. Chitosan’s ability to quickly gel relies on the formation of inter- and intramolecular cross linkages between TPP phosphates and chitosan amino groups. The properties of prepared CS nanoparticles depends on physicochemical conditions such as pH, temperature, time, and functionalization or modification by specific ligands [111,112].

It is widely known that CS NPs are able to encapsulate various compounds. Glutaraldehyde crosslinked chitosan showed promising adsorption ability for AFL B1 (73%), OTA (97%), ZEN (94%), and FUM 1 (99%) but no obvious adsorption for DON and T2 (<30%) in a buffer system simulating gastrointestinal conditions [113]. Although having great binding capacity, glutaraldehyde is considered to be toxic. LD_50_ for rats has been determined to be 1.30 mL 50% a.i./kg body wt. On the other hand, TPP is a nontoxic, anionic chelating agent forming stable CS NPs. Using a water-in-oil microemulsion encapsulation method, magnetic Fe_3_O_4_ CS NPs were synthetized as patulin adsorbents with high magnetic properties, adsorption capabilities, and hypotoxicity. Patulin molecules were adsorbed completely after 5 h with an adsorbent concentration of 400 μg in conditions mimicking the pH of juice. In vitro cytotoxicity and acute toxicity tests showed negligible cytotoxicity, no toxic response, or histopathology in treated mice [114].

#### Nanoclay Binders

This group includes minerals that are used for the detoxification of mycotoxins from food and feed, such as montmorillonite, bentonite, zeolite, or hydrated sodium (calcium) aluminosilicate. Their specificity is the willingness to form multiphase solid materials where one of the phases has one, two, or three dimensions of less than 100 nm, e.g., montmorillonite. Montmorillonite nanocomposite (MN) has been introduced as a perspective sorptive additive possessing sizable surface area, higher porosity, strong cation exchange activities, and more active sites, which enable its interaction with mycotoxins [115]. The in vitro obtained adsorption capacity of MN for AFL was estimated to be 66.67 μg/mg MN. In vivo testing in broilers demonstrated no toxic effect with a 3 g/kg diet [116]. Moreover, MN addition increased super oxide dismutase and glutathione peroxidase activities and simultaneously reduced malondialdehyde levels. Modified nanomontmorillonite by organic cations (cetyltrimethylammoniumbromide) increased the hydrophobicity of the surface of the mineral and showed a high affinity for AFLs, ZEA, and FUM B1 adsorption in rat models in vitro [117]. 

Unlike nanocomposites, halloysite (Al_2_Si_2_O_5_(OH)_4_) naturally occurs as a small cylinder (nanotubes) that has a wall thickness of 10–15 atomic alumosilicate sheets, an outer diameter of 50–60 nm, an inner diameter of 12–15 nm, and a length of 0.5–10 μm [118]. Its outer surface is mostly composed of SiO_2_ and the inner surface of Al_2_O_3_, and, hence, those surfaces are oppositely charged [119]. The external siloxane and internal aluminol surface of halloysite is efficient as an absorbent (described above) or could be used as a carrier for drugs [120,121,122,123], nucleic acids [124], antibacterial agents [125], and antioxidants [119,126,127,128,129,130]. Therefore, halloysite is an efficient adsorbent both for cations and anions. Moreover, halloysite nanotubes could be modified by various surfactants to enhance their sorption properties and specificity [131]. For instance, stearyldimethylbenzylammonium chloride has been shown to improve protection efficacy against the detrimental effects of ZEN exposure [132]. The ZEN sorption properties in vitro by modified halloysite nanotubes were investigated from simulated gastric fluid and simulated intestinal fluid. Adsorption efficiency for ZEN was estimated to be 1 mg/mL after 120 min. The results indicated mitigated toxic and estrogenic effects of ZEN, including changes in oxidative stress biomarkers and organ weights in rat animal model [133]. Promising results have been published in another study that focused on a comparison of ZEN toxicity with and without halloysite addition and its impact on swine kidney. ZEN treatment significantly increased biochemical parameters, inflammatory cytokines, and degenerative changes in the kidney and induced oxidative damage in plasma. Moreover, the addition of halloysite in combination with ZEN induced a re-establishment of biochemical parameters, plasma oxidative stress enzyme activities, and the normal histology of the kidney [134]. An implication of this is the possibility that halloysite is a prospective mycotoxin adsorbent due to its safety for micro-organisms, worms, fish, and small animals [135,136].

#### Prospective Use as Antibodies

Nanomaterials are not only promising sorbents but are also able to couple different molecules such as antibodies or targeting peptides. The application of antibodies has been highly developed in the field of medicine and target drug therapy [137,138]. Briefly, there are three ways of antibody conjugation on the surface of NPs: by adsorption, by direct covalent linkage between the surface of the NP and the antibody, or by using adapter molecules such as streptavidin and biotin. Direct coupling via adsorption requires specific surface properties which allow the interaction with antibody. Preferably, covalent attachment of the antibody allows interaction with antigen binding sites. For this purpose, carboxylic acids, which is the most common group present on NP surfaces (especially carbon nanostructures), could be employed for reaction with ligands. The prerequisite for effective antibody binding is the production of a stable association, a controlled amount of immobilized antibody, and the preservation of antibody activity [139,140,141,142]. 

In recent years, there has been increasing interest in sample pretreatment using antibody-decorated magnetic nanoparticles. This technique would facilitate purification by magnetism and combine a high capacity for purification with fast dispersibility due to the small size of the particles. It was estimated that during a 5-min purification step, an average of 80% of the ZEN and AFL B 1 returns from mycotoxin-contaminated feed mixture (10–50 ng/mL) was achieved [143]. Nanobodies (Nb) are derived from heavy chains of antibodies. Magnetic beads carrying poly (acrylic acid) brushes (MB@PAA) were fabricated as an “Nb container” for improving AFL B1 adsorption capacity. The achieved binding capacity of 0.23 mg·g^−1^ was reached [144]. In the case of magnetic separation of mycotoxins, there is no need to restrict this to chemically synthetized nanoparticles, as natural maghemite nanoclusters produced by magnetotactic bacteria can be employed with satisfactory results [145,146]. On the surface of magnetosomes, antimycotoxin antibodies can be easily attached as well as on chemically synthetized nanoparticle [147]. Future research should therefore concentrate on the investigation of the biological synthesis of nanoparticles for magnetic separation of mycotoxins from food and feed. 

### 3.3. Nanomaterials that Impact Mycotoxin Elimination from Affected Organisms 

Previous studies have reported that actual exposure to mycotoxins leads to higher oxidative stress in organisms [148,149]. Because an effective antidote does not exist, nutritional supplements could suppress the toxic impact of mycotoxin, decrease tissue damage caused by oxidative stress, and allow the body to sustain a viable immune system that can eradicate the pathogen. For instance, higher doses of natural antioxidants such as tocopherol, selenium, or zinc are recommended in animal feed [150,151]. In recent years, it was found that cerium, zinc (titanium) oxide, silver, gold, selenium, or carbon-based nanoparticles show unique antioxidant properties [152,153,154]. Moreover, better antioxidant properties of metal nanoparticles could be reached by green synthesis. It is well known that green-synthetized nanoparticles are enriched with natural compounds, which leads to their better donor activity [155]. Although their effectiveness against mycotoxin intoxication has been not explored yet, their beneficial impact on organisms should be assumed.

**Figure 3:**
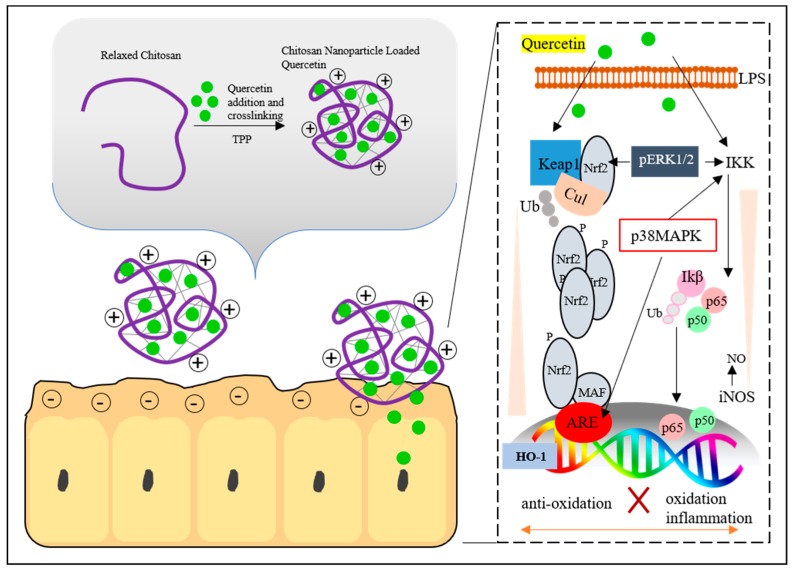
Scheme of the quercetin (Q)-loaded chitosan (CS) nanoparticle mechanism of action. The relaxed polysaccharide structure of the chitosan is crosslinked with tripolyphosphate (TPP). The Q is entrapped in the CS structure. The positive charge of NPs electrostatically interacts with the negatively charged epithelial cell wall. Quercetin triggers a hepato-protective cascade that leads to antioxidant protection via stimulation of nuclear factor E2-related factor 2 (Nrf2)-induced heme-oxygenase-1 (HO-1) production. Transcriptional response is mediated by the acting element termed (ARE) found in the promoters of genes encoding the detoxication enzymes. Q-inhibited lipopolysacharide (LPS) induced nitric oxide synthase (iNOS) and NO production via IκB kinase (IKK) and p38 mitogen-activated protein kinases (p38MAPK). The proposed mechanism of action of quercetin is adapted from [166], with permission from Public Library of Science, 2015.

Polymeric nanocapsules are able to protect and deliver antioxidants to the target tissue. Ideally, the compound is transported to the target site where it is gradually released from the transporter. The protective role of chitosan nanoparticles singly, plus quercetin (Q) at low (140 mg/kg b.w.) or high (280 mg/kg b.w.) doses, was investigated against OTA-induced toxicity on mice. Q is a member of the flavonoids family and it is well known that Q has wide bioactivity, including hypolipidemic and antioxidant properties. Previous studies have reported that Q showed a protective effect against the toxic effect of mycotoxins such as aflatoxin [156,157], OTA [158,159,160], T2 [161], zearalenone [162,163], and alternariol [164]. Round-shaped nanoparticles consisting of quercetin showed a protective effect on OTA-treated rats (3 mg/kg diet). CS-NPs/Q-supplemented animals showed significant improvements in food intake, body weight gain, serum biochemical parameters, histological picture and histochemical analyses, antioxidant enzymes such as glutathione peroxidase (GPx), super-oxide dismutase (SOD) and catalase (CAT) and malondialdehyde (MDA), DNA fragmentation, and gene expression due to minimal changes in serum biochemical parameters in comparison with untreated mice models [165]. Combined treatment with Q and CS NPs enhanced the ability to donate Q protons. Figure 3 presents a possible mechanism of action of Q-loaded CS nanoparticles via the Keap1-Nrf2 pathway (200 nm).

## 4. Efficiency Evaluation of Nanoparticles as a Solution for Eliminating the Risk of Mycotoxins

The issue of nanomaterials has received considerable critical attention regarding their toxicity on the living organisms. In addition, the massive increase of nanotechnology in many applications has caused the emergence of an opposing discipline (nanotoxicology). It could be assumed that newly synthetized nanoparticles are immediately examined for their toxicity and applicability. Nanotoxicity depends on particle solubility, surface area, number of particles per volume, surface charge, size, and tendency to agglomerate, which determine the elimination nanoparticles from the body [167]. The mentioned properties indicate the mechanism of action. Among those that have been described are the interaction with cell membrane, apoptosis induction, ROS production, inhibition of mitochondrial functions, lipid peroxidation, or autophagy [168]. Experimental studies have proven that nanoparticles could act as oxidative stress inductors, cytotoxically, as inflammatory agents, or could interact with nucleic acids and thereby contribute to the damage of both microorganisms and higher plants and humans [169]. Current toxicologic studies have found that the most organism-friendly are cerium oxide nanoparticles, fullerenes, or polymeric nanoparticles (Polyethylenglycol or chitosan). On the other hand, many inorganic nanoparticles have been considered as hazardous [170]. An objective assessment of toxicity seems to be difficult. A number of publications have been created on this topic and consortia and research groups have emerged as well. So, the status of this issue is still evolving. Interestingly, particle toxicity often works differently on model stem cells and organs. Furthermore, viability tests, such as the MTT assay, are sensitive to pH and medium, as found Jo et al. [171]. The relationship between the dose and the response of the organism must be taken into account as well as the accumulation in the environment and the chronic exposure of the organism to low doses [172,173,174].

In terms of efficiency evaluation, the toxicity of antimycotoxin nanomaterials and the toxic effect of mycotoxins have to be carefully considered. A key problem with nanomaterial efficiency against mycotoxin evaluation is the uncomplicated studies that are unilaterally focused. In reviewing the literature, no relevant data was found on the association between nanomaterial effectivity, toxicity, and the received dose of mycotoxins from food and feed. There are predominantly studies that focus on nanoparticle synthesis. Large-scale production has been improved by the fact that the prices of nanoparticles (listed in Table 3) are comparable to those of commonly used bentonite for the elimination mycotoxins from feed (2–8 USD per gram in the Czech Republic). Therefore, our intention was to outline the relationship between the threat of concentration of mycotoxins taken from food and the effectiveness of nanoparticles to eliminate them (Table 3). Nanoparticle efficiency against mycotoxins (AFL, OTA) vary from 0.065 to 1000 μg/mg depending on the nanomaterial used (magnetic graphene oxide < maghemite and halloysite nanoparticles < nanodiamonds < montmorillonite nanocomposite < chitosan nanoparticles). The scientific literature shows the toxic effect of the most common mycotoxins to be in the range of 1–30 mg/kg of the feed dose [175]. Theoretically, only 30 mg of nanoparticles per 1 kg of compound feed is sufficient to eliminate the toxic effect. In the case of the toxicity of selected nanoparticles, we have found from the literature that the safe level for mice is on average 0.3–16,000 mg/kg. Taken together, the results of nanoadsorbent efficiency against mycotoxins would have been far more persuasive if the authors had considered the actual practical feed dose of mycotoxins. 

## 5. Conclusions

In this review, we summarized the current findings of mycotoxins and their elimination by nanoparticles. Many researchers see the application potential of nanomaterials in the field of biosensors for mycotoxin detection rather than elimination. Although the exceptional properties of nanomaterials have obliged the development of this field of knowledge, the combination of knowledge of disciplines from toxicology, nanotechnology, and agricultural practices is on the rise. These findings further support the idea of the one nanoparticle which could be employed for mycotoxin detection as well as elimination and could serve as a nanocage for nutrition delivery. Further research should be done to investigate the detailed toxicity and efficiency testing as is also required in other nanotechnology disciplines. The conclusion of this review suggests that nanomaterials have interesting adsorption properties, which make them promising for mycotoxin elimination.

## Figures and Tables

**Table 1:** Antifungal nanoparticles synthetized by the chemical route.

Organism	Type of Particles	Inhibition Dose	Average Size	Reference
*Alternaria brassicicola*	Silver nanoparticles	100 ppm	NA	[56]
*Alternaria solani*	Silver nanoparticles	10 ppm	14 nm	[57]
*Alternaria solani* and *Sclerotium rolfsii*	PEGylated Mancozeb	3 mg/L	NA	[58]
*Aspergillus flavus*	Thyme essential oils in chitosan-benzoic acid nanogel	300 mg/L	100 nm	[59]
*Aspergillus flavus*	Menthe piperita essential oils in chitosan-cinnamic acid nanogel	500 ppm	100 nm	[60]
*Aspergillus flavus*	Cuminum cyminum essential oils in chitosan-caffeic acid nanogel	650 ppm	100 nm	[61]
*Aspergillus flavus*	Nanodispersed Cinnamaldehyde by surfactant (Tween 80)	1.0 mM	60 nm	[62]
*Aspergillus flavus*	Silver nanoparticles	5 μg/mL	4.5 nm	[63]
*Aspergillus flavus*	Titanium dioxide nanoparticles	1 g/L	30 nm	[64]
*Aspergillus niger*	Silver-, copper-, and nickel-based nanoparticles	65 μg/mL	15 nm	[65]
*Aspergillus niger*	Pullulan and silver nanoparticles	1.7 mg/g	9 nm	[66]
*Aspergillus parasiticus*	Citrate-coated silver nanoparticles	50 ng/mL	20 nm	[67]
*Aspergillus parasiticus*	Silver nanoparticles	180 μg/mL	NA	[68]
*Fusarium culmorum*	Silver nanoparticles	20 mg/L	35 nm	[69]
*Fusarium graminearum*	Chitosan nanoparticles	5000 ppm	200 nm	[70]
*Fusarium oxysporum*	Chitosan silver nanocomposites	100 μg/mL	370 nm	[71]
*Fusarium oxysporium*	Alumina nanoparticles	400 mg/L	200 nm	[72]
*Fusarium solani*	Ag-doped Titan oxide nanoparticles	0.43 mg/plate	NA	[73]
*Penicillium expansum* and *Botrytis cinerea*	Zinc oxide nanoparticles	3 mmol/L	70 nm	[74]
*Penicillium digitatum* and *Fusarium solani*	Copper nanoparticles	20 and 60 μg/mL	NA	[75]
*Penicillium verrucosum*	Silica and silver nanoparticles	5–100 ppm	0.65 nm and 200 nm	[76]
*Penicillium* and *Mucor*	Zinc oxide nanoparticles	5 mg/mL	600 nm	[77]

**Table 2:** Antifungal green synthetized nanoparticles.

Organism	Mediator	Type of Particles	Inhibition Dose	Average Size	Reference
*Aspergillus* sp. and *Rhizopus* sp.	Aloe vera leaf extract	Ag	100 μL of 1 M	70 nm	[78]
*Aspergillus ochraceus*	*Aspergillus terreus* and *Penicillium expansum*	Ag	3 and 9 g/100 mL	20 nm	[79]
*Aspergillus niger* and *Aspergillus flavus*	*Cissus quadrangularis*	CuO	1000 ppm	30 nm	[80]
*Aspergillus flavus*	*Penicillium citrinum*	Ag	4000 ppm	54 nm	[81]
*Aspergillus fumigatus* and *Candida albicans*	*Bacillus* species	Se	100 μg/mL	140 nm	[82]
*Candida*, *Aspergillus*, and *Fusarium*	*Arthroderma fulvum*	Ag	1 mg/mL	15 nm	[83]
*Fusarium*	*Chaetomium globosum*	Ag	500 mg/L	15 nm	[84]

**Table 3:** Binding capacity and dose evaluated as safe in mouse models for selected mycotoxin nanoadsorbents.

Nanostructure	Preparation Method	Ref.	USD/g	Dose Evaluated as a Safe in Mouse Models	Ref.	Mycotoxin	Binding Capacity	Ref.
Nanodiamonds	ion and laser bombarding, CVD, hydrothermal, ultrasonic, electrochemical	[176]	20–100	<25 mg/kg	[177]	AFL	10 μg/mg	[108]
						OTA	15 μg/mg	
Magnetic graphene oxide	coprecipitation, covalent bonding, electrostatic self–assembly, impregnating graphene oxide with magnetic nanoparticles	[178]	8–20	0.3 mg/kg	[179]	DON, ZEA	0.065 μg/mg	[109]
Surface active maghemite nanoparticles	coprecipitation, sol–gel synthesis, microemulsion, flow injection synthesis, hydrothermal synthesis, flame spray pyrolysis, decomposition of organic precursors at high temperatures, oxidation of magnetite nanoparticles	[180]	20–100	<10 mg Fe/kg	[181]	Citrin	175 μg/mg	
Chitosan nanoparticles	ionotropic gelation, microemulsion, emulsification solvent diffusion, polyelectrolyte complex, reverse micellar method	[182]	1–5	16,000 mg/kg	[183]	AFL	0.8 μg/mg	[116]
						ZEA, OTA	1 μg/mg	
Montmorillonite nanocomposite	hot intercalation technique, in situ polymerization, solution induced intercalation 10, melt processing	[184]	1–5	1000 mg/kg	[185]	AFL	67 μg/mg	[119]
Modified halloysite nanotubes	naturally occuring	[121]	<1	10 mg/mL	[119]	ZEA	1000 μg/mg	[136]
